# P04.31. Determining attitudes and use of complementary and alternative medicine and integrative medicine amongst undergraduates

**DOI:** 10.1186/1472-6882-12-S1-P301

**Published:** 2012-06-12

**Authors:** M Broukhim, A Reeves, N Huynh, M Liu

**Affiliations:** 1 University of California, Irvine, Irvine, USA

## Purpose

The purpose of this study was to determine the perceptions, attitudes, and use of Complementary and Alternative Medicine (CAM) and Integrative Medicine (IM) in UC Irvine undergraduate students to better understand the current opinion trends and use of CAM/IM amongst students. It was expected that there would be an overall positive outlook, perception, use, and interest in CAM/IM in the current undergraduate population.

## Methods

This is a prospective, cross sectional study using an electronic questionnaire conducted on the undergraduate population at University of California, Irvine from Fall 2010 to Spring 2011. Email invitations were sent to all undergraduate students to participate in this study. All data were collected anonymously and IRB approval was secured from UC Irvine. Descriptive and comparative analysis was done on the twenty-one questions using SPSS 16. Additional support for the study was obtained from the Undergraduate Research Opportunities Program (UROP).

## Results

Out of the 23,000 undergraduates, 2,800 responded to the survey, comprising 12 percent of the campus. One-third of respondents stated that they had used CAM before. Major drives for using CAM were due to friend/relative recommendation (18.4%) followed by efficiency/effectiveness (13.1%), and curiosity (12.4%). Pre-health students’ primary preferences for taking education on CAM were classes within their major (72%), as a requirement for graduation (70%), as a holistic major or minor (53%), and outside their major (22%). It was found that only 9% of pre-health majors taking the survey were not interested in more CAM classes.

## Conclusion

We find that most students are interested in attaining more CAM education if readily available. Additionally, we found an overall interest in CAM, use of CAM modalities, and a desire for further education if provided.

